# Our Advertising Department

**Published:** 1875-02

**Authors:** 


*Our Advertising Department.*

We have always believed, and experience has only strengthened our belief, that a department of select advertisements is an indispensable feature of a medical journal. The manufacturer of pure chemicals and drugs and surgical instruments should have a direct medium for communicating knowledge of his business to the medical profession; and on the other hand, the members of the profession should be kept posted as to where they can get what they may need in their practice, as well as in all the improvements in the manufacturing of instruments, appliances, chemicals, drugs, etc. And in this respect we beg leave to say that, though the journal may not have as many subscribers as a few medical journals in the North, still its influence is second to none, circulating, as it does, over an immense territory, and penetrating to remote sections North and South. It is, therefore, unexcelled as an advertising medium, which fact all interested should not fail to remember.

Such a journal should be taken and patronized by the druggists everywhere, and medical men, desiring the success of a first-class journal in the South, should not fail to invite the attention of druggists to these important features of the RECORD.

Our readers will please call the attention of their home druggists to the advantages offered in this department.

